# Kinetic Energy Harvesting with a Piezoelectric Patch Using a Bistable Laminate

**DOI:** 10.3390/mi16040410

**Published:** 2025-03-30

**Authors:** Sonia Bradai, Slim Naifar, Piotr Wolszczak, Jarosław Bieniaś, Patryk Jakubczak, Andrzej Rysak, Grzegorz Litak, Olfa Kanoun

**Affiliations:** 1 Measurement and Sensor Technology, Technische Universität Chemnitz, Reichenhainer Straße 70, 09126 Chemnitz, Germany; sonia.bradai@etit.tu-chemnitz.de (S.B.); olfa.kanoun@etit.tu-chemnitz.de (O.K.); 2 Laboratory of Electromechanical Systems, National School of Engineers of Sfax, University of Sfax, Sfax 3038, Tunisia; slim.naifar@enis.tn; 3 Higher Institute of Applied Sciences and Technology of Gabès, University of Gabès, Gabès 6029, Tunisia; 4 Department of Automation, Lublin University of Technology, Nadbystrzycka 36, 20-618 Lublin, Poland; p.wolszczak@pollub.pl; 5 Institute of Technology and Information Technology, Zamojska Academy, Pereca 2, 22-400 Zamość, Poland; 6 Department of Materials Engineering, Lublin University of Technology, Nadbystrzycka 36, 20-618 Lublin, Poland; j.bienias@pollub.pl (J.B.); p.jakubczak@pollub.pl (P.J.); 7 Department of Computerization and Robotization of Production, Lublin University of Technology, Nadbystrzycka 36, 20-618 Lublin, Poland; a.rysak@pollub.pl

**Keywords:** bistable laminated plate, optical fibers, piezoelectric, composite materials, energy harvesting

## Abstract

A bistable effect on a laminate structure with a piezoelectric patch was tested to harvest kinetic energy. The composite bistable plate was prepared in the autoclave with two different orientations of the glass fibers. The dynamic tests were performed on the universal testing machine using cyclic vertical compression excitation. During the tests, the bottom edge of the plate was clamped firmly while its upper edge was attached with some clearance to enable sliding. In such a configuration, the friction force between the plate and upper clamp element is responsible for the plate excitation. Simultaneously, the plate has enough space to change the shape between the two equilibria. During the harmonic excitation of the testing machine operating mode, a piezoelectric element was placed on the bistable plate and its voltage and normalized power outputs were evaluated. The experiments were repeated with additional mass distribution, which influenced the natural frequency of the plate.

## 1. Introduction

Structural multistability is an interesting nonlinear property that has attracted much attention in the field of energy harvesting [1]. The transition from one stable state to another is usually associated with moderate or large displacement (deformation), which is beneficial for piezoelectric energy harvesting. The switch passes through the potential well, and it is characterized by the sudden change of shape, causing the immediate release of accumulated energy.

Due to the existence of multiple solutions, multistable properties can be used in the frequency broadband energy harvesting effect to improve the efficiency of vibration energy harvesting [2,3,4,5]. A highly interesting approach to bistability is in laminate structures [6,7,8,9,10,11,12,13]. A dynamic response of a bistable shell structure with the snap-through phenomenon was studied experimentally and theoretically in the context of energy harvesting [6]. The forcing was realized in the system as a seismic mass excitation where variable inertial force exited the structure, leading to the switch between two stable shapes [7,8,9,10] for a large enough amplitude of excitation. The corresponding structures are based on at least two layers that are characterized by minimum energy, including the initial residual stress implemented in the manufacturing process.

Several attempts at energy harvesting based on transducers with bistable laminate structures [10,14,15] have been reported. Consequently, Piezoelectric elements were attached to the laminate surface to generate electrical energy via the direct piezoelectric effect during plate deformation, including dynamic snap-through between the two stable states as a result of external mechanical excitations. Piezoelectric energy harvesting can be used for various applications, ranging from powering small electronic devices and wireless sensor networks to more specific applications like healthcare applications [16]. Among notable examples of bistable laminate energy harvesters, a significant contribution was reported in [8]. The authors used a 200 × 200 mm bistable laminate plate made of Carbon Fiber Reinforced Polymer (CFRP) with inertial masses attached to each corner. Four PZT-5A flexible piezoelectric patches were glued to the surface of the plate using a high shear strain epoxy. During the experimental investigations, the tested plate was mounted from its center to an electromechanical shaker. The results showed the frequency dependency of the snap-through effort. In terms of harvested power, 34 mW and 27 mW were obtained for an acceleration forcing level of 2 g in response to intermittency and large amplitude limit cycle oscillations, respectively.

In the next report [14], Bett et. al. studied the optimal configurations for a bistable laminate energy harvester based on four piezoelectric patches on the top surface, and four piezoelectric patches oriented perpendicularly on the bottom surface, each positioned at the center of one quarter of the laminate surface. An investigation made into the plate, which was held by each of its four corners, demonstrated that increasing the piezo-element size increases the area from which electrical energy can be harvested and therefore increases the harvested energy. However, a threshold is observed where the laminate stiffness increases and hence the deflection and electrical energy drop.

Most researchers harvesting energy from plate-type structures seek to maximize the energy harvested by increasing the piezo-element size to cover most of the plate area [12,13]; however, a study of the position effect of the piezo element was not systematically considered.

In this paper, we propose to investigate the energy harvesting performance of bistable laminate structures as a function of the orientation and position of the piezoelectric patch. An analysis of the deformation of the plate was first determined in order to find the suitable position of the plate. Additionally, in order to apply mechanical excitations to the plate with the minimum possible number of constraints, i.e., without fixing the plate from its center or corners, a universal tensile machine was employed to make a controlled deflection.

In this way, the external energy is absorbed by the plate in this phase of the excitation process. Large enough stress with associate bending leads to stronger deformation, and it can overcome the potential barrier between the characteristic stable shapes. In this situation, we observe snapthrough. This effect can give a short primary impulse of voltage output on the attached piezoelectric patch accompanied by a number of secondary damped oscillations.

## 2. Preparation of the Bistable Plates

The laminate plates for current investigation are conventional composite bistable plates (G1) based on glass fiber-reinforced polymer (GFRP) and a modified version of bistable composite based on the FML (fiber metal laminates) concept (GM1). The bistable plates are manufactured by stacking alternating layers with a thickness of unidirectional R-glass fiber-reinforced thermosetting epoxy resin prepregs (Hexcel, USA). The nominal fiber content is about 60% of the total volume. The selected strength properties of the glass fiber-reinforced polymer used in the tested bistable plates are presented in Table 1. In the case of the GM1 plate, the additional metal layer-based aluminum 2024-T3, with 0.3 mm thickness, is integrated with the composite layers. The aluminum layer was selected due to its excellent compatibility with glass fiber composites when properly treated with chromic acid anodizing (CAA), ensuring the structural integrity of the FML [17,18,19]. Additionally, it adds ductility and impact resistance while maintaining a relatively low weight penalty, which affects the dynamic characteristics of the response during snap-through events. This comparison provides insights into potential design trade-offs between harvesting efficiency and structural properties for different application requirements. Table 2 presents configurations of tested composite bistable plates.

**Figure 1:**
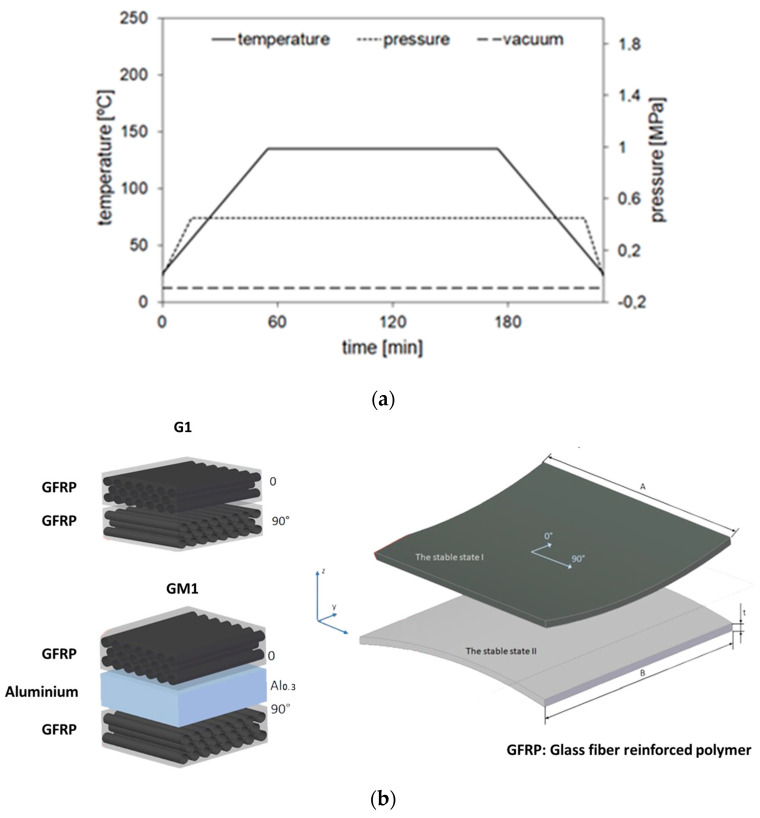
(**a**) Diagram of the curing process in autoclave experimental bistable plates; (**b**) structure and equilibrium states of the two bistable plates.

The composite and FML bistable plates are produced in the Department of Materials Engineering at Lublin University of Technology by the autoclave method using a Scholz Maschinenbau autoclave system (Scholz Maschinenbau GmbH, Coesfeld, Germany). The cure cycle is carried out at a heating rate of 2 °C/min up to 135 °C and held at this temperature for 2 h. The pressure and the vacuum used are 0.4 and 0.08 MPa, respectively. The diagram of the curing process in the autoclave of experimental bistable plates is shown in Figure 1.

## 3. Experimental Investigation

### 3.1. Experimental Setup

**Figure 2:**
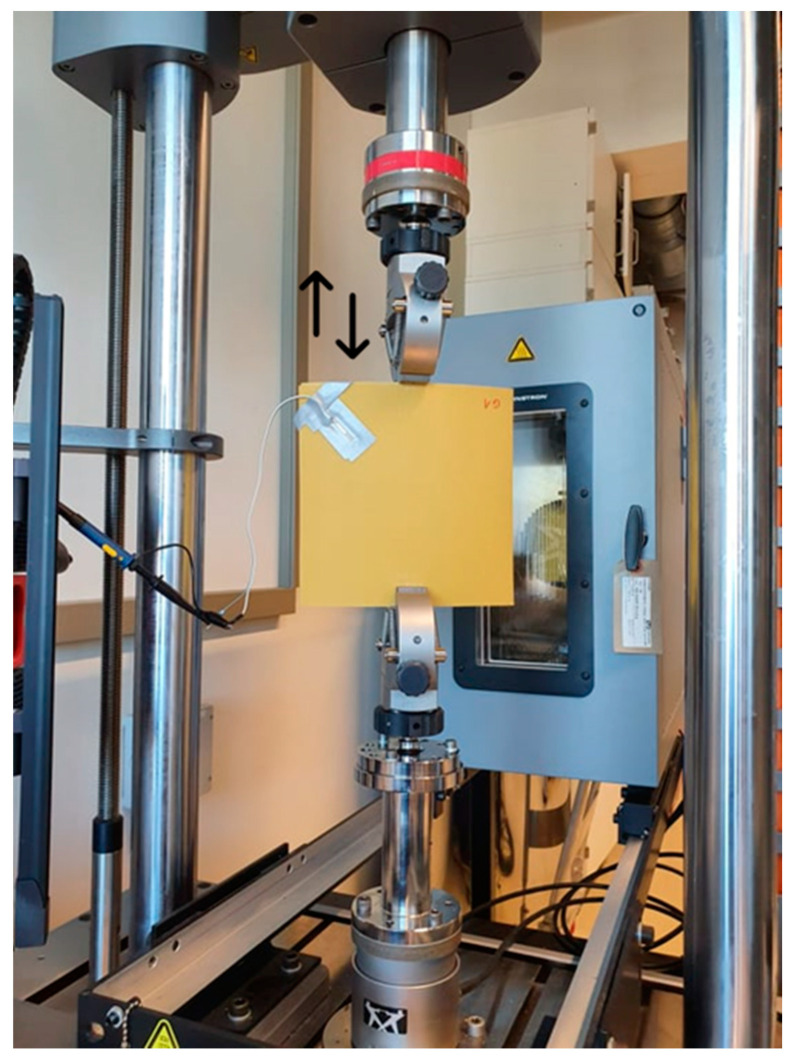
G1 bistable plate fixed at the universal testing machine.

The experimental evaluation of the piezoelectric energy harvester (PEH) implemented on the bistable plates is conducted in order to study several parameters, in particular, applied displacement frequency, piezoelectric element orientation, and additional mass effect. For this purpose, the bistable plate is fixed to a universal testing machine (Figure 2), and a harmonic displacement with an amplitude of 2 mm is applied on it with different frequency levels, varying from 0.5 Hz to 2 Hz with a step of 0.5 Hz.

The bistable plate is clamped firmly from the bottom, and its upper edge is attached with some clearance to enable sliding. In this configuration, the clamp of the universal testing machine maintains contact with the upper edge of the plate while allowing some freedom of movement. The 2 mm displacement amplitude was specifically selected to ensure reliable snap-through transitions between the two stable states of the plate. In such a configuration, the harmonic force between the plate and upper clamp element of the universal testing machine leads to the plate excitation and hence the switch between its two stable positions under the harmonic applied excitation. The piezoelectric element is fixed to one corner of the bistable plate, as presented in Figure 2.

### 3.2. Evaluation of the Displacement at the Level of the Plate

**Figure 3:**
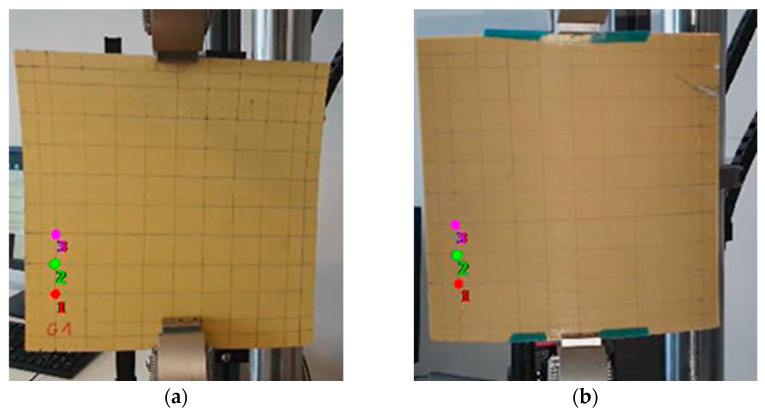
Experimental setup for the evaluation of the position change during the execution of the two stable positions for the plate (for two different deformations (**a**,**b**)). Note the marker positions and sketched rectangular lattice for deformation identification.

This section aims to evaluate the displacement through the bistable plates while switching between both stable positions. This is executed by evaluating the position change of markers on the bistable plate by recording a video showing both bistable positions (Figure 3).

**Figure 4:**
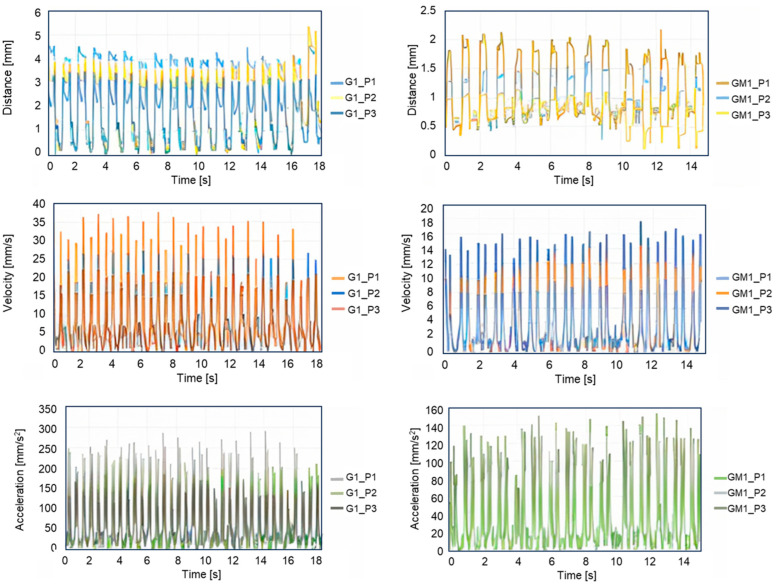
Evaluation of the displacement, velocity, and acceleration at the different locations of the bistable plates G1 and GM1 on the left and right hand sides, respectively.

In the present study, the evaluation of the executed displacement at each marker from 1 to 3 is conducted. For the markers 1, 2, 3 on the G1 plate, maximum displacements of 4.73 mm, 5.61 mm, and 3.54 mm were reached, while for plate GM1, displacements of 2.17 mm, 1.65 mm, and 1.43 mm were recorded (Figure 4), respectively. The evaluation of displacement at different positions on the plate surface was specifically conducted to identify regions of significant deformation during snap-through events. As shown in Figure 4, marker 2 exhibited the highest displacement (5.61 mm for G1 plate), indicating a region of maximum deformation.

Based on these displacement measurements, we positioned the piezoelectric element near high-strain regions to investigate energy harvesting potential. Similarly, the magnet positions (P1–P5) were strategically selected to evaluate how added mass affects the strain distribution and natural frequency of the plate. Our subsequent analysis revealed that positions P1 and P2 provided superior performance for the concentric orientation, while P2 showed the highest output for the radial orientation. This systematic examination enables the identification of optimal configurations for piezoelectric element placement to maximize energy harvesting. It is important to note that both the location and orientation of the piezoelectric patch significantly impact performance, with their relative effectiveness being frequency-dependent, as discussed in the following section.

### 3.3. Effect of the Piezoelectric Direction on the Output Voltage

**Figure 5:**
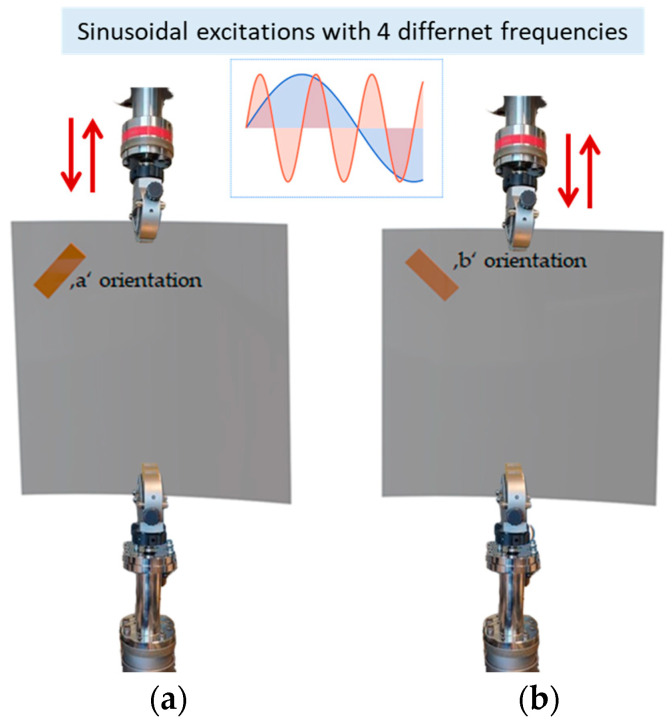
Orientations investigated for the piezoelectric element on the bistable plate (**a**) concentric position: ‘a’ orientation; (**b**) radial position: ‘b’ orientation.

The aim of this section is to evaluate the output voltage of the piezoelectric element due to its orientation. The same piezoelectric element was used throughout all experiments to ensure consistent electrical characteristics. Voltage measurements were performed under open-circuit conditions. This consistent measurement approach was sufficient to evaluate and compare the relative performance of different configurations, as our primary focus was on identifying optimal piezoelectric placement and orientation rather than absolute power optimization. Figure 5 shows the two orientations that are evaluated for the piezoelectric element at the same position. The experiments are executed for an applied displacement with a frequency varying from 0.5 Hz to 2 Hz with a step of 0.5 Hz, and the piezoelectric element response is recorded through a PICOSCOPE 2205A.

**Figure 6:**
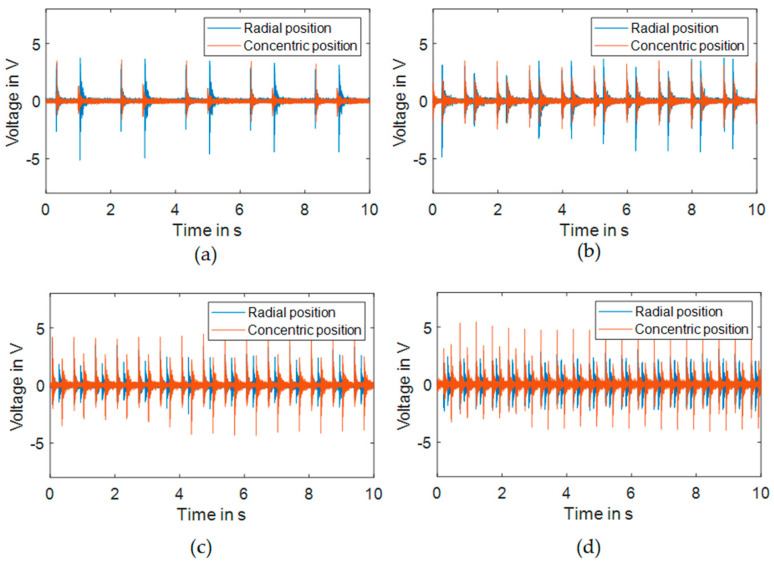
Response of the piezoelectric element for the two orientations relative to the bistable plate for selected excitation frequencies (**a**) 0.5 Hz, (**b**) 1 Hz, (**c**) 1.5 Hz, and (**d**) 2 Hz.

For the same orientation and different frequencies, the RMS value of the amplitude is presented (Figure 6). Note that the RMS value is proportional to the amount of harvested energy. The RMS value behavior varies with excitation frequency and depends significantly on the piezoelectric orientation and stable position configuration. Our frequency analysis shows that for the first stable position, the concentric orientation demonstrates progressively increasing RMS values with higher frequencies, reaching maximum values at 2 Hz. The radial orientation exhibits higher RMS values at lower frequencies but decreases as frequency increases. In the second stable position, similar trends are observed but with different magnitudes. The alignment between piezoelectric element orientation and principal strain directions during snap-through events creates these frequency-dependent characteristics. The strain distribution patterns during transitions between stable states interact differently with each orientation depending on the frequency, with concentric orientation capturing strain more effectively at higher frequencies and radial orientation performing better at lower frequencies. These findings emphasize the importance of matching piezoelectric orientation to the expected operational frequency range to optimize energy harvesting performance.

**Figure 7:**
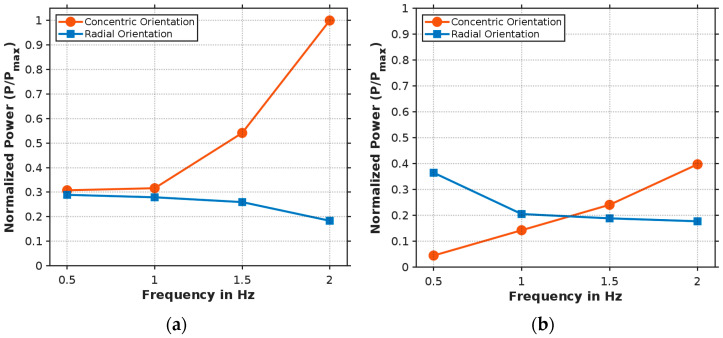
Normalized power output vs. excitation frequency for (**a**) first stable position and (**b**) second stable position.

The normalized power output relative to the excitation frequency for both piezoelectric orientations reveals significant frequency-dependent behavior (Figure 7). In the first stable position (Figure 7a), the concentric orientation exhibits a pronounced increase in power output with frequency, reaching maximum normalized power at 2 Hz, while the radial orientation shows a steady decline to approximately 18% of maximum power at 2 Hz. For the second stable position (Figure 7b), the concentric orientation demonstrates consistent growth from minimal power at 0.5 Hz to approximately 40% at 2 Hz, whereas the radial orientation decreases from 36% to 18% across the frequency range, with both orientations producing equal power at approximately 1.25 Hz. These opposing trends indicate that strain distribution patterns during snap-through events differ significantly between stable positions, with higher frequencies increasingly favoring the concentric orientation. This frequency-dependent behavior highlights the importance of piezoelectric orientation selection based on the operational frequency range and predominant stable position for optimal energy harvesting efficiency.

### 3.4. Effect of Additional Mass on the Output Voltage

**Figure 8:**
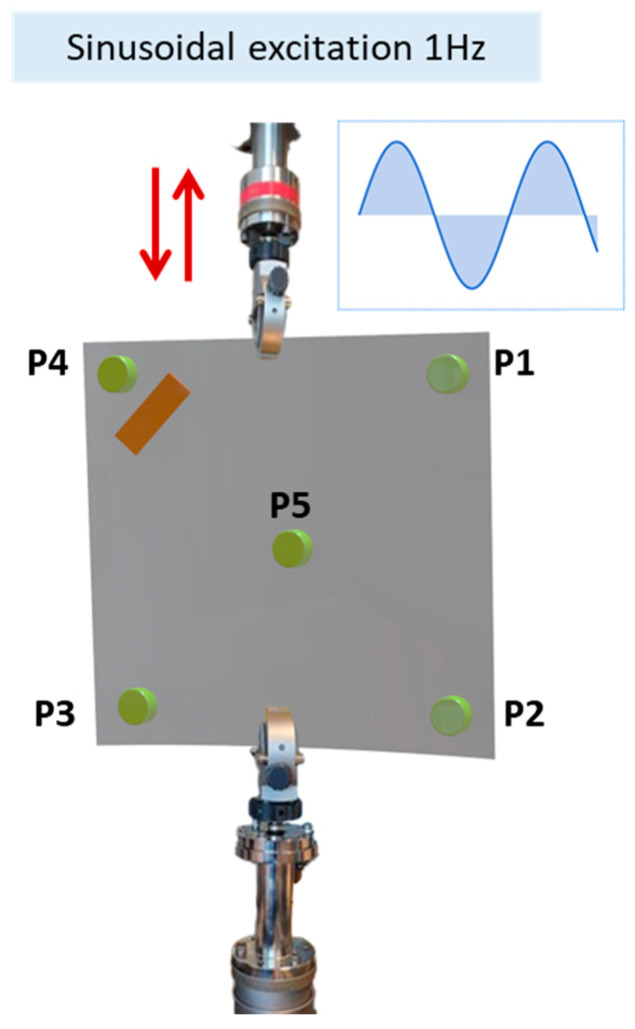
Added mass position through the bistable plate. Magnets were placed on both sides of the plate with attractive orientations.

Continuing the study, we considered additional mass effects with regard to the dynamical plate response. In particular, a magnetic mass (disc magnet of 10 mm diameter and 3 mm height) of about 2 g is added to the plate at five different positions P1 to P5, as shown in Figure 8.

**Figure 9:**
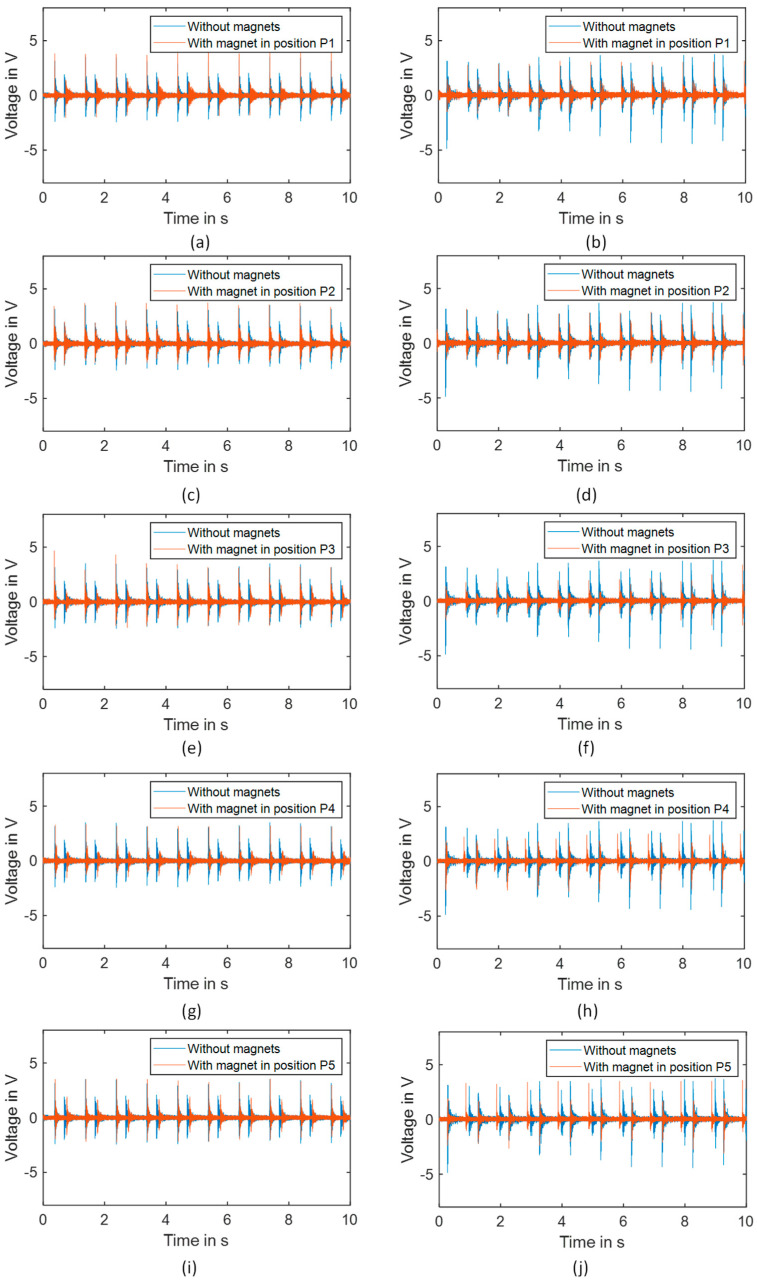
Response of the piezoelectric element relative to the different mass positions for 1 Hz for different added mass positions for concentric piezoelectric harvester orientation at positions (**a**) P1, (**c**) P2, (**e**) P3, (**g**) P4, and (**i**) P5, and for radial piezoelectric harvester orientation at positions (**b**) P1, (**d**) P2, (**f**) P3, (**h**) P4, and (**j**) P5.

Figure 9 and Table 3 present the voltage responses of the piezoelectric element for different added magnet positions at 1 Hz excitation frequency. The voltage patterns (Figure 9) vary significantly depending on both the magnet position (P1–P5) and the piezoelectric orientation (concentric vs. radial). For the concentric orientation (left column), position P1 exhibits higher voltage peaks for the two stable positions. For the radial orientation (right column), positions P1, P2, and P5 demonstrate higher voltage amplitudes compared to positions P3 and P4. The time interval between voltage peaks also varies with the magnet position, indicating changes in the dynamic response and transition time between stable states. This position-dependent behavior confirms that the strategic placement of additional mass can effectively tune the harvesting performance by altering the strain distribution and dynamic characteristics of the bistable plate.

**Figure 10:**
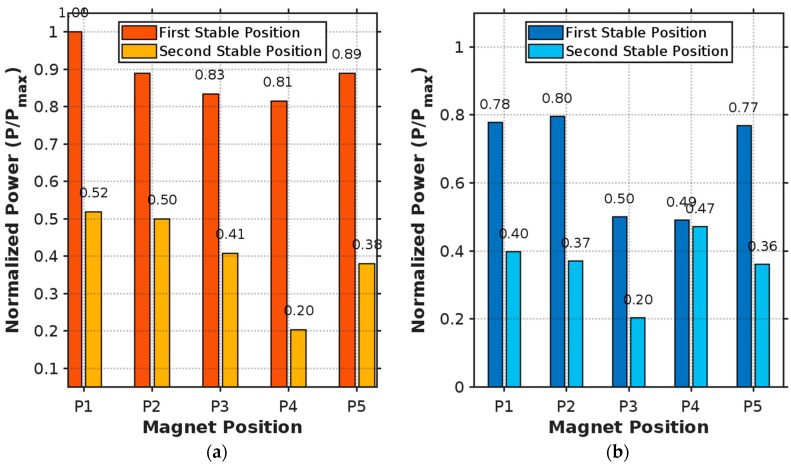
Normalized power output for different magnet positions: (**a**) concentric orientation and (**b**) radial orientation.

The results showed that the additional mass influences both the resonance frequency of the plate and the amplitude of oscillations depending on the selected positions. Figure 10 presents the normalized power output for different magnet positions in both orientations. For concentric orientation, position P1 yields the maximum power output (1.00) in the first stable position, while all other positions (P2–P5) show slightly lower but still significant performance (0.81–0.89). In the second stable position, P1 and P2 provide the highest output (0.52 and 0.50, respectively), with P4 showing the lowest (0.20).

## 4. Conclusions

We presented the concept of implementing a piezoelectric energy harvester on a bistable composite structure. For this purpose, two plates type are investigated in this paper, which are G1 and GM1. The bistable plates are realized based on glass fiber-reinforced polymer (GFRP) G1 and a modified version of bistable composite based on FML (fiber metal laminates) concept (GM1). A comparative study between both plates’ behavior is conducted to characterize the bistable behavior and the effect of the addition metal layer through the composite. The piezoelectric element generates the most significant response during fast snap-through motion.

Furthermore, piezoelectric harvester performance was investigated for different configurations. The results showed that orientation and placement significantly affect voltage output, with frequency-dependent behavior observed between concentric and radial orientations. Concentric orientation demonstrates superior power output at higher frequencies, while radial orientation performs better at lower frequencies, with performance varying between stable positions. Additional mass placement near the piezoelectric element increased oscillation amplitude. The snap-through mechanism in bistable plates provides an effective energy harvesting approach, where energy accumulates through residual stress and releases rapidly during deformation.

While this study provides valuable insights into piezoelectric orientation and added mass effects, future research should examine additional parameters such as the size and shape of the piezoelectric elements and magnet geometry for further optimization. The advantage of using bistable plates is the snap-through stimulation vibrations of the system. In such a process, the energy is slowly accumulated due to residual stress and released quickly during deformation.

## Figures and Tables

**Table 1:** Selected strength properties of the glass fiber-reinforced polymer in the tested bistable plates.

Tensile Strength [MPa]		Young’s Modulus [GPa]		Poisson’s Ratio		Shear Strength [MPa]	Shear Module [GPa]
0°	90°	0°	90°	0°	90°	±45°	±45°
1534	75	46	15	0.27	0.09	58	5.23

**Table 2:** Configuration of tested composite bistable plates.

Composite Bistable Plates	Dimensions (a × b) [mm]	Layers Configurations	Plates Thickness (t) [mm]
G1	200 × 200	0/90	0.5
GM1	200 × 200	0/Al/90	0.8

**Table 3:** Statistics of voltage amplitudes (response of the piezoelectric element) relative to the different mass positions, cyclic activation frequency, and positions of magnet.

Piezolayer Orientation	Cyclic Activation Frequency	Location of Magnet	Amplitude Statistics		
			Average	Maximum	Standard Deviation
Concentric	0.5 Hz	P1	0.1045	4.8842	0.2015
	1 Hz	P1	0.1247	4.3558	0.2174
	1.5 Hz	P1	0.1391	4.9333	0.2370
	2 Hz	P1	0.1729	4.8445	0.3143
Concentric	1 Hz	P1	0.1247	4.3558	0.2173
		P2	0.1339	3.7783	0.2353
		P3	0.1206	4.6667	0.2166
		P4	0.1101	3.6006	0.1899
		P5	0.1136	3.6895	0.1976
Radial	1 Hz	P1	0.1438	3.3189	0.2218
		P2	0.1451	3.14097	0.2406
		P3	0.1202	3.853	0.1846
		P4	0.147	2.7335	0.253
		P5	0.1255	3.7642	0.2261

## Data Availability

Data are available on request.
